# Cross-Sectional Study on the Prevalence of PCV Types 2 and 3 DNA in Suckling Piglets Compared to Grow–Finish Pigs in Downstream Production

**DOI:** 10.3390/pathogens11060671

**Published:** 2022-06-10

**Authors:** Matthias Eddicks, Roland Maurer, Pauline Deffner, Lina Eddicks, Wolfgang Sipos, Sven Reese, Vojislav Cvjetković, Roman Krejci, Tanja Opriessnig, Mathias Ritzmann, Robert Fux

**Affiliations:** 1 Clinic for Swine at the Centre for Clinical Veterinary Medicine, Ludwig-Maximilians-Universität München, 85764 Oberschleissheim, Germany; r.maurer@med.vetmed.uni-muenchen.de (R.M.); p.deffner@med.vetmed.uni-muenchen.de (P.D.); m.ritzmann@lmu.de (M.R.); 2 Institute of Veterinary Pathology at the Centre for Clinical Veterinary Medicine, Ludwig-Maximilians-Universität München, 80539 Munich, Germany; lina.eddicks@patho.vetmed.uni-muenchen.de; 3 Clinical Department for Farm Animals and Herd Management, University of Veterinary Medicine Vienna, 1210 Vienna, Austria; wolfgang.sipos@vetmeduni.ac.at; 4 Institute for Anatomy, Histology and Embryology, Ludwig-Maximilians-Universität München, 80539 Munich, Germany; s.reese@anat.vetmed.uni-muenchen.de; 5 Ceva Tiergesundheit GmbH, 40472 Duesseldorf, Germany; vojislav.cvjetkovic@ceva.com; 6 Ceva Santé Animale, 33500 Libourne, France; roman.krejci@ceva.com; 7 The Roslin Institute and The Royal (Dick) School of Veterinary Studies, University of Edinburgh, Easter Bush, Midlothian EH25 9RG, UK; tanja.opriessnig@roslin.ed.ac.uk; 8 Department of Veterinary Diagnostic and Production Animal Medicine, College of Veterinary Medicine, Iowa State University, Ames, IA 50011, USA; 9 Division of Virology, Department of Veterinary Sciences, Ludwig-Maximilians-Universität München, 80539 Munich, Germany; robert.fux@lmu.de

**Keywords:** porcine circoviruses, oral fluids, suckling piglets, tissue samples

## Abstract

Vertical transmission is a consistently discussed pathway of porcine circovirus type 2 (PCV2) and porcine circovirus type 3 (PCV3) transmission in pigs. To evaluate the presence of PCV2 and PCV3 in piglets, we collected tissue samples from 185 piglets that were crushed within the first week of life from 16 farms located in Germany and Austria. Pooled samples consisting of thymus, inguinal lymph node, myocardium, lung and spleen were examined for PCV2 and PCV3 by qPCR. Furthermore, oral fluid samples (OFS) from grow–finish pigs were collected and examined the same way. In piglets, PCV2 was highly prevalent (litters: 69.4%; piglets: 61.6%), whereas PCV3 prevalence was low (litters: 13.4%; piglets: 13.0%). In total, 72.6% and 67.2% of all collected OFS were PCV2 or PCV3 positive, respectively. Sow vaccination against PCV2 was identified as a protective factor concerning PCV2 in piglets (OR: 0.279; CI: 0.134–0.578; *p* < 0.001), whereas the porcine reproductive and respiratory syndrome virus (PRRSV) vaccination of sows was identified as a protective factor concerning PCV3 in piglets (OR: 0.252 CI: 0.104–0.610; *p* = 0.002). Our results show that PCV2, but not PCV3, is ubiquitous in suckling piglets and that early PCV3 infections might be modulated by PRRSV–PCV3 interaction. However, the ubiquitous nature of both viruses in older pigs could be confirmed.

## 1. Introduction

Porcine circovirus type 2 (PCV2) is an economically and health-relevant pathogen in the domestic pig population that is associated with several disease syndromes (porcine circovirus diseases; PCVD) affecting growing pigs as well as breeding animals [1]. In breeding herds, piglets can be infected with PCV2 horizontally via shedding of infectious virus particles by the dam or neighboring sows [2,3], or vertically via diaplacental intrauterine infection [3,4]. The relevance of the intrauterine way of transmission was demonstrated in several experimental and field studies over the last two decades. Whereas results from North America and Brazil showed high amounts of PCV2 DNA-positive sera from newborn piglets [5,6,7], results from Europe exhibit contrary results [8,9], which may be due to the differences of PCV2 infection dynamics and PCV2 vaccination status at the same time of sample collection. However, it is undisputed that the diaplacental transmission of PCV2 plays a crucial role in the spread of PCV2 within the domestic pig population and, among others, could lead to latent PCV2 infections [10]. Recent study results from a subclinically PCV2-infected German multiplier herd indicate the relevance of PCV2 viremia in the early stage of gestation for this way of transmission, particularly concerning young sows [11]. Furthermore, Dvorak et al. [5] demonstrated that besides the diaplacental route of transmission, the early infection of piglets can also occur due to environmental PCV2 contamination in the farrowing pen. The chance for piglets to become infected with PCV2 in the early stages of life was further elucidated by a study on in vivo-derived porcine morulae- and blastocyst-stage embryos [12]. Specifically, it was shown in an experimental study design that zona pellucida-free morulae and blastocysts are susceptible to PCV2 infection. In a Spanish field study, the positive effects of PCV2 vaccination in a subclinically infected breeding herd included improved pig vitality and higher numbers of live-born piglets, which further supports the hypothesis that PCV2 infections during gestation occur regularly [13]. Taken together, there is a vast number of possibilities for piglets to become infected with PCV2 in the early stages of life.

Concerning porcine circovirus type 3 (PCV3), similar to PCV2, associations of this virus with different diseases or clinical outcomes in pigs have been proposed, including porcine dermatitis and nephropathy syndrome (PDNS) [14], porcine respiratory diseases complex (PRDC) [15], congenital tremor [16] or reproductive disorders [17,18]. Experimental proof of these associations is still lacking for PCV3, however, the detection of PCV3 DNA in aborted fetuses [17,18] indicates a diaplacental route of infection and subsequent PCV3 transmission to naïve littermates in the early stages of life. Furthermore, Kedkovid et al. [19] demonstrated that in analogy to PCV2 [20], PCV3 DNA can also be detected in sow colostrum.

Based on the current knowledge of early PCV2 and PCV3 infections, we hypothesized that the horizontal transmission of PCV3 to littermates is a frequent event in early life, but also in later stages of production. The present study was designed to evaluate the detection rates of the two virus species in suckling piglets under field conditions. Specifically, tissue samples from piglets that were crushed by their mothers within the first week of life were collected and analyzed, while oral fluid samples (OFS) were collected from older pigs. Utilizing crushed piglets enabled a purposeful sampling of different tissues with respect to “the three Rs” (Replacement, Refinement and Reduction) desired for scientific working with animals to avoid animal experiments and to limit the number of animals and their suffering, as published in Directive 2010/63/EU of the European Parliament and of the Council of 22 September 2010 on the protection of animals used for scientific purposes.

## 2. Results

### 2.1. Presence of PCV2 and PCV3 DNA in Suckling Piglets

PCV2 DNA-positive suckling piglets were present on 15 of 16 farms (Germany 13/13, Austria 2/3). In total, 64.9% (95% CI: 56.6–73.1%) of all litters and 61.6% (95% CI: 55.1–68.6%) of the individual piglets were PCV2 DNA-positive, respectively. PCV3 was detected in tissue samples from 9/16 farms (8/13 Germany; 1/3 Austria). In 13.4% (95% CI: 7.9–19.5%) of all litters and 13.0% (95% CI: 8.1–17.8%) of the individual piglets, PCV3 DNA was present. Dual PCV2 and PCV3 infection in the tissue samples of the individual suckling piglets was detected in 7.0% (95% CI: 3.8–10.8%). Chi^2^ testing revealed that significantly more litters and significantly more piglets were PCV2- than PCV3-positive (*p* < 0.001). The number of PCV2 and PCV3 DNA-positive litters, piglets and OFS for each individual farm is shown in Table 1.

### 2.2. Qualitative Evaluation

Dichotomous variables were first checked for significant associations using the chi^2^ test. Significantly associated factors (*p* ≤ 0.05) were subsequently included in a binary logistic regression. The results concerning PCV2 and PCV3 DNA-positive tissue pools are shown in Table 2, Table 3 and Table 4. In brief, PCV2 sow vaccination was a protective factor concerning the presence of PCV2 DNA in piglets while sow vaccination against the porcine reproductive and respiratory syndrome virus (PRRSV) was identified as a protective factor for the presence of PCV3 DNA in piglets. On-farm replacement of gilts tended to be negatively associated with numbers of PCV2 DNA-positive piglets.

### 2.3. Quantitative Evaluation in Tissue

To assess whether the variables from the binary logistic regression model also affect the viral load in the samples, we conducted a generalized mixed model that included the same independent variables as the regression and additionally considered double infection with PCV2 and PCV3. For this examination, only PCV2, PCV3 and PCV2 and PCV3 DNA-positive samples were included. This examination revealed that neither for PCV2 nor for PCV3 do any of the factors significantly influence the Cq value in the tissue samples.

**Figure 1:**
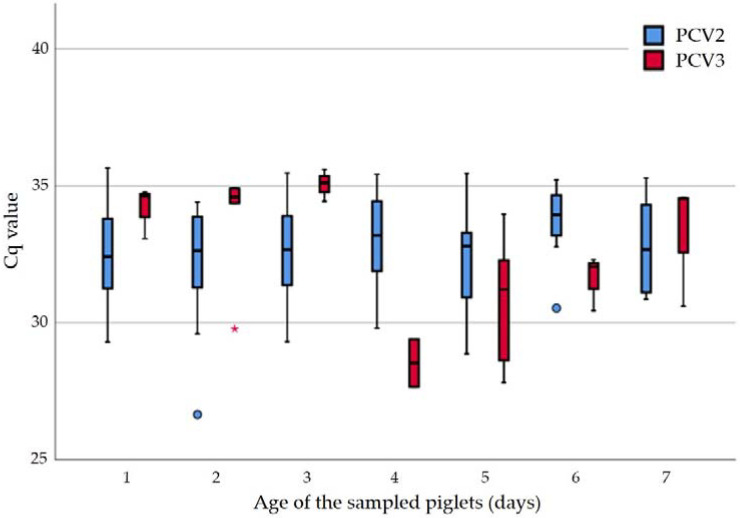
Cq values of tissue pools (PCV2 and PCV3 PCR) in regard to the age (1–7 days of age) of the sampled piglets (circle and asterisk indicate outliers).

The further statistical analysis included examinations concerning the relationship (Spearman’s rho) between the quantitative outcome (Cq values) of the PCRs for tissue sample pools for PCV2 and PCV3 and the factors “age of the sampled piglet”, “bodyweight of the sampled piglet”, and “parity of the corresponding dam” or “life-borne piglets in the corresponding litter”, “dead-borne piglets in the corresponding litter” and “bodyweight of the sampled pig”. These examinations revealed that the viral load of PCV3 in the tissue pools increased with the increasing age of the sampled piglets (r_s_: −0.413; *p* = 0.045). In the generalized mixed model, this observation turned to a tendency (*p* = 0.058). No further significant correlation was observed. The Cq values for the tissue pools under consideration of the age of the sampled piglet are shown in Figure 1.

### 2.4. Presence of PCV2 and PCV3 DNA in OFS of Finisher Pigs

**Figure 2:**
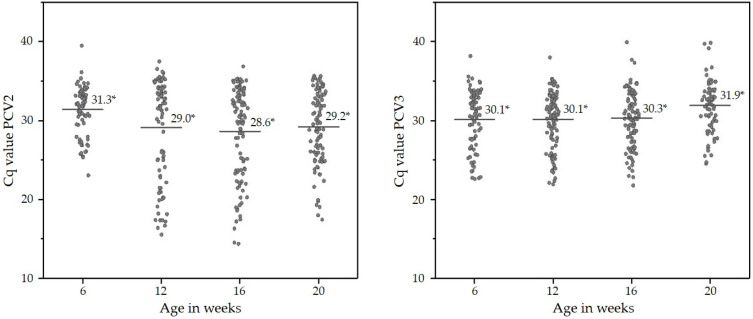
Cq values of single PCV2 or PCV3 DNA-positive OFS of growing and fattening pigs at different times of sampling. Each dot represents the Cq value of a single OFS. Horizontal lines represent the median Cq value at the particular time of sampling (* mean Cq value).

In grow–finish pigs, pen-based OFS were used to check for PCV2 and PCV3 DNA. All but one farm (15/16) were positive for PCV2 and PCV3 based on the PCR results of the OFS. The examination of the OFS independently from the time of sampling revealed that 72.6% (95% CI: 69.3–76.3%) and 67.2% (95% CI: 62.9–71.1%) were PCV2 or PCV3 DNA-positive, respectively. In total, 51.1% (95% CI: 46.4–55.3%) of the OFS were positive for both viruses. The qualitative results of the molecular examinations of the OFS with respect of the time of sampling are shown in Table 5. An overview on the Cq values of all positive OFS are given in Figure 2.

## 3. Discussion

The present study examined the occurrence of PCV2 and PCV3 DNA in suckling piglets crushed by their mothers within their first week of life, and includes the results of the PCV detection in OFS in downstream production steps. The rationale for this kind of sampling was to bring our study in line with the “the three Rs” desired for scientific working with animals, as published in Directive 2010/63/EU of the European Parliament and of the Council of 22 September 2010 on the protection of animals used for scientific purposes. Although this type of sampling has its limitations, it enabled us to gain relevant and interesting data. However, the number of PCV2 or PCV3 DNA-positive piglets might be overrepresented, as it cannot be excluded that the detected pathogens impacted the health of the piglets, making them more prone to being crushed. While this may be true for individual piglets, it is not necessarily applicable to an entire farm. However, the high number of farms with PCV2-(15/16) and PCV3-(9/16) positive litters indicates an overall high prevalence of PCV2 or PCV3 in breeding farms. While high prevalence rates of PCV2-positive suckling piglets have been reported before [5,6], this is one of the first reports on the occurrence of PCV3 in suckling piglets. Concerning PCV2, our findings are in contrast to our own results [8] and results from colleagues from the Netherlands [9]. However, this difference can be best explained by the differences in sample type among the studies. Whereas pre-suckle sera were examined in the previously mentioned studies, we used tissue samples, as viremia might not necessarily be present in PCV2-infected piglets [10]. Furthermore, pre-suckle sera are a good measure to evaluate the number of viremic piglets at the time of birth, but the time span of one week used in the present study also includes the possibility of infection after birth. This topic was discussed in detail by Dvorak et al. [5], who showed that piglets can be readily infected with PCV2 in utero and that they are under the constant challenge of PCV2 after birth through contact with infected sows and a contaminated farrowing environment. Moreover, even colostrum can be PCV2-positive [20,21] and may possibly lead to early PCV2 infections in suckling piglets [20]. The presence of PCV3 DNA in the colostrum samples of sows was also readily reported by Kedkovid et al. [19]. In the aforementioned study, high PCV3 loads in colostrum samples were associated with high viral loads in the corresponding sows. As we did not collect blood samples from the sows, we cannot refer to this observation. Interestingly, the number of PCV3 DNA-positive piglets was significantly lower than PCV2 DNA-positive ones. While the high PCV2 DNA detection rate confirms the ubiquitous nature of PCV2 and the results from other colleagues [5,6], PCV3 seems not to be ubiquitous in that age group. Referring to this, an interesting aspect of our study results is the observation of tendentially increasing viral loads in correlation with the progressed age of the sampled piglets. Although our study has a cross-sectional character, these findings indicate that early infections might be of relevance for the spread of PCV3 within the following production steps as the increasing viral loads give a hint to viral replication. On the other hand, co-infections with both viruses did not lead to higher viral loads in the tissue samples of the piglets, which indicates that the infection of a pig with both viruses does not enhance replication at that age. Overall, the viral loads in the tissue samples appeared low range, indicating subclinical disease rather than PCV-associated reproductive failure [1,22].

In grow to finish pigs, the prevalence for both PCV2 and PCV3 was high, which is in line with several other field studies [23,24,25]. Interestingly, in all but one farm, PCV2 DNA and PCV3 DNA were present in the grow–finish phase. Furthermore, a high proportion of the OFS was positive for both PCV2 DNA and PCV3 DNA.

In our study, we identified PCV2 sow vaccination as a protective factor concerning the number of PCV2 DNA-positive piglets up to one week of age, whereas the PRRSV vaccination of sows seems to have a protective impact concerning the number of PCV3 DNA-positive piglets. Whereas the protection against PCV2 after sow vaccination against the latter is in line with the results of Oliver-Ferrando et al. [13], who were able to show positive effects concerning the viability and reproductive performance of piglets and sows, respectively, the lack of any effect on the prevalence of PCV3 DNA-positive piglets indicates no cross-protection against PCV3 after sow vaccination against PCV2. This is in line with previous observations in German fattening farms, where no significant correlation between PCV3 detection and the PCV2 vaccination status of the herds was observed [23]. Furthermore, it has been demonstrated that the PCV3 ORF2, coding for the immunodominant capsid protein, shows no significant similarity with the corresponding ORF2 of most other circoviruses, including PCV1 and PCV2 [14,26,27]. Interestingly, sow vaccination against PRRSV was significantly associated with a lower number of PCV3 DNA-positive piglets. These results indicate a possible role of PRRSV as a co-factor in case of early PCV3 infections in piglets and might be correlated with the immunosuppressive nature of PRRSV [28], which is antagonized by the specific vaccination measure. However, although this observation was significant, there is a need for further elucidation under controlled or more standardized conditions.

## 4. Material and Methods

### 4.1. Farms and Animals

The study was carried out on 16 farms between July 2018 and August 2019. The participation was voluntarily. The study took place in several federal states of Germany and Austria. The farms were either conventionally farrow-finish farms (14/16) or farrow-to-wean farms directly connected to a fattening farm (2/16). Farm details were recorded by a questionnaire at the time of sample collection and are available in the supplementary materials (Table S1). All farms fulfilled the legal requirements concerning the housing conditions of the corresponding countries.

### 4.2. Collected Materials

#### 4.2.1. Tissues

Participating farmers were asked to collect piglets that were crushed by their dams within the first week of life. In total, 185 crushed piglets from 134 litters were obtained. The piglets were stored frozen at −18 °C on the farms until collection by the staff of the Clinic for Swine of the LMU Munich when OFS and farm-specific data were also collected. On arrival at the Institute for Veterinary Pathology of the LMU Munich, the piglets were defrosted and tissue samples were collected and processed. The collected tissues included inguinal lymph node, spleen, thymus, lung and myocardium. The samples were assigned to two aliquots (one diagnostic and one back-up sample).

#### 4.2.2. Oral Fluid Samples (OFS)

Oral fluid samples were collected in a cross-sectional manner from pigs at the age of 6, 12, 16 and 20 weeks at each farm. One cotton rope (IDEXX, Westbrook, ME, USA) was used to sample 20 pigs. In total, we collected OFS from approximately 200 pigs per farm and age group. If more than 20 pigs were housed in one pen, two ropes were placed. In total, 515 OFS were collected, resulting in a total sampled population of maximal 10,300 individuals from 16 farms. The number of collected OFS per farm is available from Table 1. OFS collection took place as described by Prickett et al. [29]. The collection period lasted 25 to 30 min; afterwards, the lower, wet part of the rope was inserted into the supplied plastic bag and squeezed manually to release the oral fluid. The sampled OFs were decanted into supplied 5 mL centrifuge tubes and were centrifuged at 1560 g for 10 min to separate feed particles and other contaminants from the liquid. They were stored at −20 °C until further analysis.

### 4.3. Molecular Biological Examinations

All OFS and tissue samples were examined for PCV2- and PCV3-specific DNA. For DNA isolation, the DNA Mini Kit (Qiagen) was used according to the manufacturer’s instructions. For virus detection, we used a quantitative duplex PCR (Virotype^®^ PCV2/PCV3, INDICAL BIOSCIENCE GmbH, Leipzig, Germany).

### 4.4. Statistics

Statistical calculations were performed with the software IBM SPSS Statistics version 28.0.1.0 for Microsoft^®^ Windows. The prevalence was specified as absolute and relative frequencies in percent with the 95% confidence interval. The significance level was 0.05.

Dichotomous variables were analyzed for possible associations with the PCR outcome (positive/negative) by a chi^2^ test. In case more than one independent variable was associated with the dependent variable, a binary logistic regression was also conducted. An overview of the factors and distribution of the examined population used is available as supplementary material (Table S2).

Independent factors that were significantly associated within the chi^2^ test were also used to evaluate whether these factors not only had an influence on the qualitative outcome concerning the PCV2 and PCV3 PCR, but also on the quantitative outcome (Cq values of PCV2 and PCV3 PCR in tissue pools). Therefore, we conducted a multifactorial generalized mixed linear model (gamma regression as target distribution) and added the independent factor “both viruses in the same tissue pool”. For these examinations, only PCR-positive tissue samples were included.

To assess whether the quantitative PCR outcome was potentially associated with any external factors, we conducted a bivariate non-parametric correlation according to Spearman, including “age of the sampled piglet”, “bodyweight of the sampled piglet” and “parity of the corresponding mother”. The results were verified in a multifactorial analysis, performing the generalized mixed linear model (gamma regression as target distribution) including the individual farms as random factors. Furthermore, we checked whether the Cq values had any impact on the dependent variables “life-borne piglets in the corresponding litter”, “dead-borne piglets in the corresponding litter” and “bodyweight of the sampled pig”, also by bivariate non-parametric correlations. For these examinations, only PCR-positive tissue samples were included.

## 5. Conclusions

The present data confirm the ubiquitous nature of PCV2 in suckling piglets and grow–finish pigs. In contrast, for PCV3, this observation was only true for grow–finish pigs, whereas the PCV3 prevalence was low in suckling piglets. Co-infections with PCV2 and PCV3 in the same animals were of low prevalence and had no significant effect on the viral loads or other parameters checked in this study. Sow vaccination against PCV2 was identified as a protective factor concerning PCV2, but not for PCV3, indicating no cross-protection of PCV2 vaccination against PCV3. On the other hand, the vaccination of sows against PRRSV was a protective factor concerning the PCV3 detection rate in suckling piglets, indicating that early PCV3 infections might be modulated by this specific pathogen interaction.

## Figures and Tables

**Table 1:** PCV2 and PCV3 DNA-positive litters, piglets, and OFS on each farm (n/%).

	Suckling Piglets (Up to 1 Week of Age)				Grow–Finish Pigs (6–20 Weeks of Age)	
Farm	Tissue Pools by Litters		Tissue Pools for Each Piglet		Oral Fluids	
	PCV2 % (n)	PCV3 % (n)	PCV2 % (n)	PCV3 % (n)	PCV2 %(n)	PCV3 % (n)
1	41.7% (5/12)	8.3% (1/12)	38.5% (5/13)	7.7% (1/13)	100.0% (32/32)	65.6% (21/32)
2	68.8% (11/16)	6.3% (1/16)	66.7% (12/18)	5.6% (1/18)	60.0% (21/35)	65.7% (23/35)
3	75.0% (6/8)	37.5% (3/8)	77.8% (7/9)	33.3% (3/9)	63.9% (23/36)	30.6% (11/36)
4	87.5% (14/16)	0.0% (0/16)	78.6% (22/28)	0.0% (0/28)	63.9% (23/36)	22.2% (8/36)
5	42.9% (3/7)	28.6% (2/7)	44.4% (4/9)	22.2% (2/9)	62.2% (23/37)	100.0% (37/37)
6	75.0% (3/4)	0.0% (0/4)	75.0% (6/8)	0.0% (0/8)	53.1% (17/32)	87.5% (28/32)
7	100.0% (9/9)	0.0% (0/9)	92.3% (12/13)	0.0% (0/13)	42.9% (15/35)	62.9% (22/35)
8	100.0% (8/8)	12.5% (1/8)	75.0% (12/16)	6.3% (1/16)	96.9% (31/32)	81.3% (26/32)
9	89.5% (17/19)	5.3% (1/19)	85.7% (18/21)	4.8% (1/21)	29.4% (10/34)	73.5% (25/34)
10	80.0% (4/5)	100.0% (5/5)	72.7% (8/11)	100.0% (11/11)	57.1% (16/28)	92.9% (26/28)
11	50.0% (1/2)	0.0% (0/2)	50.0% (1/2)	0.0% (0/2)	100.0% (36/36)	61.1% (22/36)
12	50.0% (3/6)	50.0% (3/6)	36.4% (4/11)	27.3% (3/11)	100.0% (38/38)	94.7% (36/38)
13	11.1% (1/9)	11.1% (1/9)	8.3% (1/12)	8.3% (1/12)	100.0% (24/24)	95.8% (23/24)
14	33.3% (1/3)	0.0% (0/3)	33.3% (1/3)	0.0% (0/3)	40.0% (8/20)	0.0% (0/20)
15	0.0% (0/7)	0.0% (0/7)	0.0% (0/7)	0.0% (0/7)	87.0% (20/23)	39.1% (9/23)
16	33.3% (1/3)	0.0% (0/3)	25.0% (1/4)	0.0% (0/4)	100.0% (37/37)	78.4% (29/37)
Total	64.9% (87/134)	13.4% (18/134)	61.6% (114/185)	13.0% (24/185)	72.6% (374/515)	67.2% (346/515)

**Table 2:** Results of chi^2^ test and binary logistic regression of dichotomous variables for PCV2 (only significant results shown here).

Independent Variable	Dependent Variable	*p*-Value Chi^2^ Test	*p*-Value Binary Logistic Regression	OR	Upper CI	Lower CI
PCV2 sow vaccination	PCV2 DNA- positive piglet	<0.001	0.001	0.279	0.134	0.578
*Mycoplasma* *hyopneumoniae* sow vaccination		0.004	0.464	-	-	-
Own replacement gilts		0.007	0.056	-	-	-

**Table 3:** Results of chi^2^-test and binary logistic regression of dichotomous variables for PCV3 (only significant results shown here).

Independent Variable	Dependent Variable	*p* Value Chi^2^ Test	*p*-Value Binary Logistic Regression	OR	Upper CI	Lower CI
Porcine reproductive and respiratory syndrome virus sow vaccination	PCV3 DNA- positive piglet	0.001	0.002	0.252	0.104	0.610
*Actinobacillus* *pleuropneumoniae* sow vaccination		0.048	0.184	-	-	-

**Table 4:** Percentage and number of PCV2 or PCV3 DNA-positive piglets with respect to PCV2 or PRRSV sow vaccination.

PCV2 Sow Vaccination	PCV2 DNA-Positive Piglets % (n)	PRRSV Sow Vaccination	PCV3 DNA-Positive Piglets % (n)
Yes	37.5% (CI: 22.2–53.7%) (15/40)	Yes	8.1% (CI: 3.7–13.2%) (11/135)
No	68.3% (CI: 60.8–75.7%) (99/145)	No	26.0% (CI: 14.9–38.5%) (13/50)

**Table 5:** Number and percentage of PCV2 or PCV3 DNA-positive OFS or both of all farms at different times of sampling.

Week of Life	PCV2 DNA- Positive OFS % (n)	95% Confidence Interval %	PCV3 DNA- Positive OFS % (n)	95% Confidence Interval %	PCV2 + PCV3 DNA-Positive OFS % (n)	95% Confidence Interval %
6	65.6% (82/125)	56.8–73.6	65.6% (82/125)	56.8–73.6	44.8% (56/125)	36.0–53.6
12	67.2% (90/134)	59.0–74.6	75.4% (101/134)	68.7–82.8	56.0% (75/134)	47.8–63.4
16	79.2% (103/130)	71.7–86.2	70.8% (92/130)	63.1–78.5	56.9% (74/130)	48.5–65.4
20	78.6% (99/126)	71.4–85.7	56.3% (71/126)	47.6–64.3	46.0% (58/126)	37.3–54.8

## Data Availability

The data presented in this study are available on reasonable request from the corresponding author.

## Supplementary Materials

The following supporting information can be downloaded at: https://www.mdpi.com/article/10.3390/pathogens11060671/s1, Table S1: Overview of the single farm specifications (ff: farrow to finish farm; Ery: *Erysipelothrix rhusiopathiae*; PPV: porcine parvovirus; IAV: influenza A virus), Table S2: Overview of independent factors used for the statistical analysis. Targets were the PCV2 and PCV3 PCR results (nominal/metric).
