# Supplementary material for: Cross-Sectional Study on the Prevalence of PCV Types 2 and 3 DNA in Suckling Piglets Compared to Grow–Finish Pigs in Downstream Production
Source: Pathogens. 2022 Jun 10;11(6):671. doi: 10.3390/pathogens11060671 (PMC9227362; doi:10.3390/pathogens11060671)
Supplement: Supplementary file 1 [file pathogens-11-00671-s001.zip › pathogens-1735517-supplementary.pdf]

pathogens

MDPI

Table S1. Overview on the single farm specifications (ff: farrow to finish farm; Ery: *Erysipelothrix rhusiopathiae*; PPV: Porcine parvovirus; IAV: Influenza A virus).

Farm	Federal state	TYPE of FARM	Number of Sows	Vaccination Sows	Vaccination Piglets	Batch System (Weeks)/Suckling Period (Days)
**1**	Bavaria	ff	180	Ery-PPV/IAV	PCV2 / M.hyo	4 / 21
**2**	Bavaria	ff	180	APP / PRRSV Ery-PPV	PCV2 / M.hyo / PRRSV	5 / 28
**3**	North Rhine-Westphalia	1:1 connected	470	M.hyo / APP / PRRSV/ IAV/ Ery-PPV	PCV2 / M.hyo / PRRSV	1.5 / 28
**4**	Lower Saxony	1:1 connected	400	PRRSV / PCV2 / Ery-PPV / IAV	PCV2 / M.hyo / PRRSV	2 / 21
**5**	Baden-Wuerttemberg	ff	360	PCV2 / PRRSV / IAV / Ery-PPV	PCV2 / M.hyo / PRRSV	1.5 / 28
**6**	Schleswig-Holstein	ff	160	M.hyo / PCV2 / PRRSV / IAV / Ery-PPV	PCV2 / M.hyo / PRRSV	1 / 28
**7**	Lower Saxony	ff	160	APP / PRRSV	PCV2 / M.hyo	3 / 28
**8**	Baden-Wuerttemberg	ff	120	Ery-PPV	PCV2 / M.hyo / PRRSV	3 / 28
**9**	North Rhine-Westphalia	ff	320	PRRSV / IAV / Ery-PPV	PCV2 / M.hyo	2 / 21
**10**	Bavaria	ff	250	Ery-PPV	PCV2 / M.hyo	1 / 28
**11**	North Rhine-Westphalia	ff	380	PRRSV / IAV / Ery-PPV	PCV2 / M.hyo	1 / 28
**12**	North Rhine-Westphalia	ff	145	M hyo / PCV2 / PRRSV / IAV / Ery-PPV	PCV2 / M.hyo / PRRSV	4 / 21
**13**	Upper Austria	ff	120	APP / M.hyo PCV2 / PRRSV Ery-PPV	PCV2 / M.hyo / PRRSV	3 / 28
**14**	Upper Austria	ff	90	PCV2 / Ery-PPV	PCV2 / M.hyo	3 / 21
**15**	Upper Austria	ff	76	Ery-PPV	PCV2 / M.hyo	3 / 28
**16**	Lower Saxony	ff	900	APP / M.hyo / PCV2 /PRRSV/ IAV/ Ery-PPV	PCV2/ M.hyo /PRRSV	1 / 28

Table S2. Overview on independent factors used for the statistical analysis. Target were the PCV2 and PCV3 PCR results (nominal/metric).

Dichotom Independent Factors	Tissue Pools in Category (Suckling Piglets)	OFs in Category (Growing-Fattening)
PCV2 sow vaccination	40/185 (21.6 %)	131/515 (25.4 %)
PCV2 piglet vaccination	185/185 (100%)	515/515 (100 %)
PRRSV sow vaccination	155/185 (73.0 %)	380/515 (73.8%)
PRRSV piglet vaccination	84/185 (45.4 %)	213/515 (41.4 %)
IAV sow vaccination	110/185 (59.5 %)	321/515 (62.3 %)
M. hyo sow vaccination	44/185 (23.8 %)	167/515 (32.4 %)
M. hyo piglet vaccination	185/185 (100 %)	515/515 (100 %)
APP sow vaccination	35/185 (18.9 %)	107 /515 (20.8 %)
APP piglet vaccination	30/185 (16.2%)	59/515 (11.5%)
Own replacement gilts	64/185 (34.6 %)	208/515 (40.4 %)
Sex of the piglets	89/185 male (48.1 %) 96/185 female (51.9 %)	Not assignable
Individual needles for each litter	40/185 (21.6 %)	132/515 (25.6 %)
